# Epidemiology of hand injuries that presented to a tertiary care facility in Germany: a study including 435 patients

**DOI:** 10.1007/s00402-022-04617-9

**Published:** 2022-09-22

**Authors:** Nicholas Moellhoff, Veronika Throner, Konstantin Frank, Ashley Benne, Michaela Coenen, Riccardo E. Giunta, Elisabeth M. Haas-Lützenberger

**Affiliations:** 1 Division of Hand, Plastic and Aesthetic Surgery, University Hospital, LMU Munich, Ziemssenstr. 5, 80336 Munich, Germany; 2 Department of Medical Information Processing, Biometry, and Epidemiology (IBE), Chair for Public Health and Health Services Research, Research Unit for Biopsychosocial Health, LMU Munich, Munich, Germany; 3 Pettenkofer School of Public Health, Munich, Germany

**Keywords:** Plastic surgery, Hand surgery, Hand trauma center, Trauma registry, Trauma surgery, Emergency department

## Abstract

**Background:**

Hand injuries compose up to 30% of all injuries in emergency care. However, there is a lack of epidemiological data reflecting patient or accident-related variables, injury types, injured anatomical structures or trauma localization.

**Objective:**

The objective of this study is (1) to provide epidemiological information on hand injuries and their patterns and (2) to visualise the frequencies of affected areas of the hand in relation to the most common trauma mechanisms using color-coded heatmaps.

**Methods:**

This prospective single-center observational trial conducted at a surgical emergency department in Germany collected data of hand trauma patients using a standardized documentation form. Demographic data, trauma-related data, diagnostic and therapeutic measures were analyzed. Color-coded heatmaps were generated marking anatomic danger zones.

**Results:**

435 patients with a mean age of 39.5 were included. Most patients admitted on their own initiative (79%). Leisure and sport injuries were most frequent (75%). Digiti II–V were injured most commonly (43%), followed by metacarpals (19%) and the thumb (14%). Blunt trauma and cuts accounted for most injuries (74%). Hand-graphics depicted color-coded frequencies of the affected areas of the palmar and dorsal aspect of the hand for the most common types of injury, as well as the most frequent circumstances of accident. Elective surgery was recommended in 25% of cases, and hand surgical follow-up was proposed in over 50% of cases.

**Conclusions:**

The dorsal aspect of the hand including the 5th metacarpal, the radial wrist and thenar region, as well as the fingertips of Digiti II/III represent anatomic danger zones to injury of the hand. Due to the large variety of potentially injured structures, diagnosis and treatment is not trivial. Specific training is required for all surgical specialties in emergency care, to increase quality of diagnostic work-up and management of hand injuries.

**Supplementary Information:**

The online version contains supplementary material available at 10.1007/s00402-022-04617-9.

## Introduction

The human hand is one of the most important tools used in daily life. Both motor skills and sensitivity are essential in order to coordinate and control the finest of movements. These movements are facilitated by the minute anatomy and the collaborative work between diverse tissues including skin, nerves, tendons and bones [1, 2]. As a “tool”, the hand is frequently exposed to daily stress and the risk of injury. Even minor injuries can cause severe functional impairments [3, 4]. Impairments restrict patients in everyday life [2], at work [5] and in societal participation [6].

There is a lack of data regarding the epidemiology of hand injuries in general. Hand injuries compose between 4 and 30% of all injuries in emergency care [7–11]. Yet, merely a few studies have been published investigating epidemiological aspects on a national and international level. Analysis often focuses on a specific anatomical structure of the hand [12, 13], on a distinct type of injury [14–16], failing to emphasize hand injuries alone [17–20] or include only a specific patient population, i.e. athletes [21–24] or children [25–27].

Hand injuries are classified according to the International Classification of Diseases (ICD-10). However, these codes are often not specific enough to adequately reflect the injured anatomical structures. In addition to this, it remains unclear whether certain patient or accident-related variables are preferably associated with certain types of injuries, anatomical structures or localizations—information which is valuable i.e. for taking preventative measures.

In order to minimize this gap, the objective of this study is (1) to provide epidemiological information on hand injuries and their patterns based on a standardized “Hand-Trauma Documentation” form utilized for the assessment of hand trauma within a single-center emergency department and (2) to visualize the frequencies of affected areas of the hand in relation to the most common trauma mechanisms using color-coded heatmaps, as a first step to determine “anatomic danger zones” of locations markedly exposed to trauma.

## Materials and methods

### Study design

The study was designed as a prospective single-center observational trial. It was conducted at a surgical emergency department of a level 1 hospital in Germany (Zentrale Notaufnahme am Campus Innenstadt, University Hospital, LMU Munich). Data were collected on attendance of the senior author (E.H.) over a 10-month period during the years 2015 to 2016. Prior to initiation, ethical approval was granted by the Institutional Review Board of the medical faculty LMU Munich (Ref.-Nr.: 184-13). The study was performed according to the principles of the Declaration of Helsinki 1996.

### Patients

Patients aged 16 years and older with any acute hand injury who presented to the surgical emergency department (University Hospital, LMU Munich) within the time period of this study, and for whom documentation with the ‘Hand Trauma Documentation’ sheet (suppl. Figure 1) was available, were included. Re-appointments were not included.

### Data collection

A ‘Hand Trauma Documentation’ sheet (suppl. Figure 1) was designed by the Division of Hand, Plastic and Aesthetic Surgery, University Hospital, LMU Munich to specifically document data of hand trauma patients. The following variables were selected from this paper-based documentation sheet: ‘Age’ (metric), ‘gender’ (female yes/no), ‘insurance status’ (private/statutory/self-payment), ‘type of admission’ (own initiative/ambulance/nursing home/referral general practitioner/other specialized medical professional/air ambulance), ‘handedness’ (right/left), ‘tetanus protection’ (yes/no), ‘circumstance of accident’ (occupational/leisure or sport), ‘injured hand’ (right/left /both), ‘localization of injury’ (thumb/finger (II–V)/metacarpal /wrist/forearm), ‘type of injury’ (blunt injury/cut/crush/needle stick/infection/burn/bite/other) ‘affected structures’ (superficial wound/bone/extensor tendon/flexor tendon/joint/nerve/muscle/vessel), ‘fracture’ (yes/no) ‘radiologic assessment’ (X-ray/computed-tomography),’Diagnosis’ (ICD-10) beside others. In addition, information was retrieved from a hand-graphic (consisting of a palmar, dorsal and skeleton illustration of the hand) which was used to mark the injured areas (see suppl. Figure 1). Any of the three illustrations of the hand could be utilized, as long as the injury was appropriately depicted. The documentation sheet was filled in with information from the examination and the case history by the attending physician and nurse and collected on days of attendance of the senior author of the manuscript (E.H.).

### Data analysis

Data were analyzed anonymously. Data are presented as means with respective standard deviation (1 SD), or as absolute and relative frequencies. All calculations were performed using SPSS Statistics 28 (IBM, Armonk, NY, USA) and Microsoft Excel (Microsoft Corporation, Redmond, WA, USA). Color-coded heatmaps were generated using a previously described method [28]. In short, physicians marked the location of injury on the hand-graphics provided within the ‘Hand Trauma Documentation’ sheet (suppl. Figure 1). Then, a grid with unique numbered cells was created and superimposed upon the marked hand-graphics. The numbered cells containing the physician´s marks were tabulated within an excel sheet. The numbered cells of all included patients were summarized, processed and visualised as color-coded heat-maps using a program written in java 1.7 (http://www.oracle.com/) utilizing methods and classes of two graphical programming libraries: Abstract Window Toolkit (Oracle Corporation, Santa Clara, CA, USA) and Swing (Oracle Corporation, Sun Microsystems, Santa Clara, CA, USA). The intensity of red color-gradation depicts the frequency of the affected locations (light—dark red corresponding to low—high frequency).

## Results

### Demographic data, patient characteristics and ways of submission into the emergency department

**Fig. 1:**
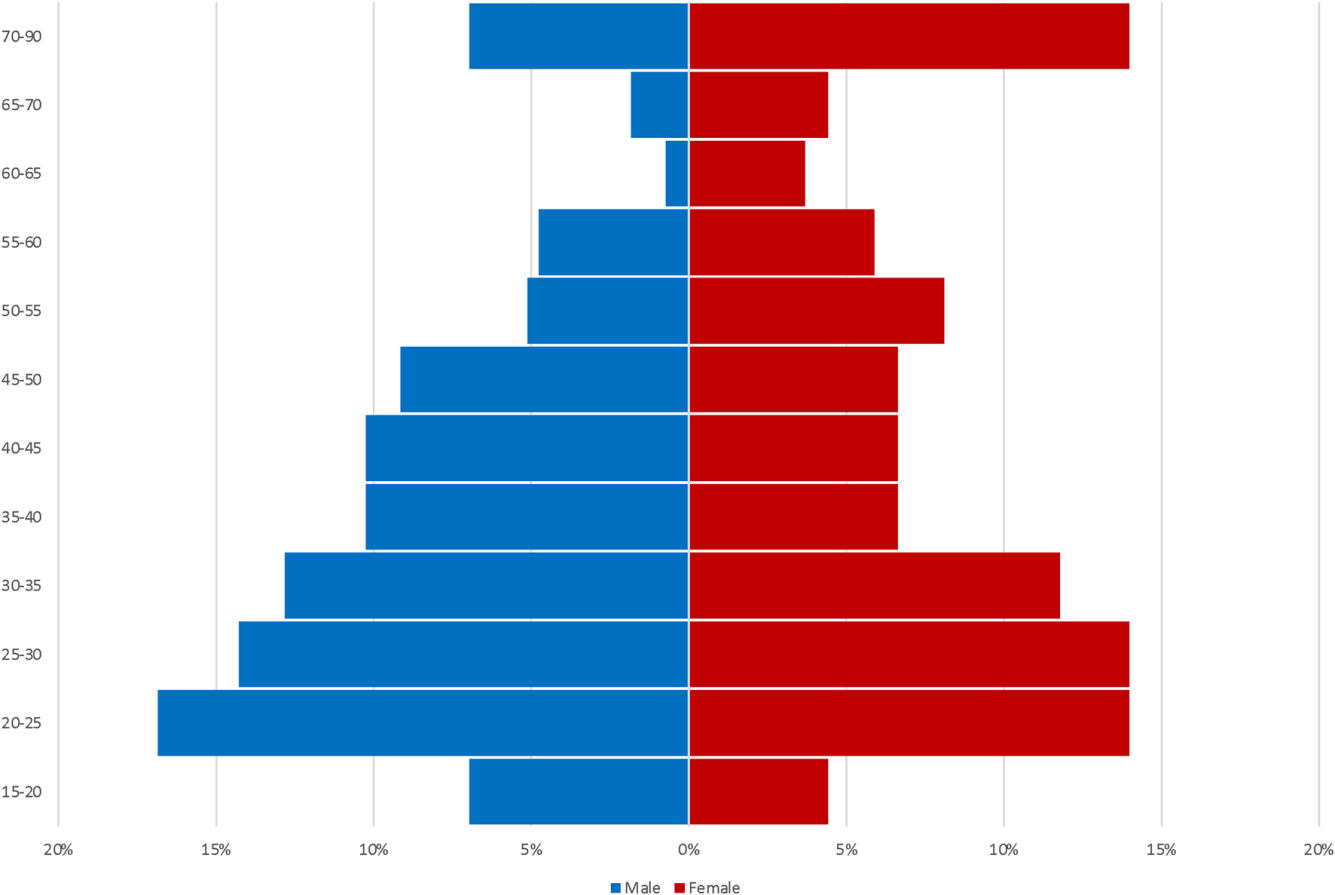
Bar graph depicting the age distribution (%) of patients with regard to male and female gender

A total of 435 patients (male 290, female 140, n/a 5) with a mean age of 39.46 ± 17.69 years (Range: 16–90) presented to the emergency department during the study interval and were included in this investigation. The distribution of age with regards to gender of patients is shown in Fig. 1. The majority of patients for whom data were available were admitted on their own initiative (79%, *n* = 106/134), followed by ambulance (16%, *n* = 21/134), referral from a general practitioner (2%, *n* = 3/134), other specialized medical professional (2%, *n* = 3/134) or air transportation (0.7%, *n* = 1/134). Of the patients for whom data were available, 69% (*n* = 100/145) were insured under a statutory insurance plan, 13% (*n* = 19/145) under a private insurance plan and 18% (*n* = 26/145) were self-paying patients.

### Trauma, circumstance of accident and type of injury

**Fig. 2:**
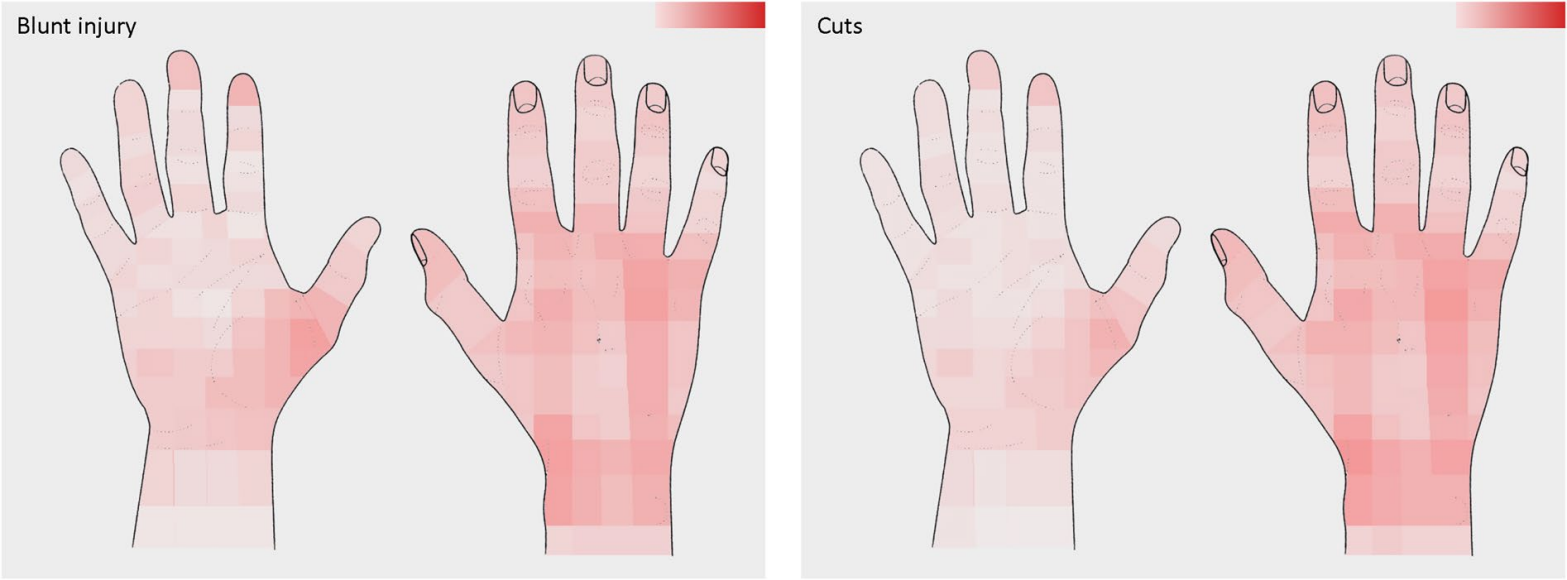
Heatmaps with color-graded frequency depicting the location of injury for blunt injury and cuts. The higher the color intensity, the higher the frequency

**Fig. 3:**
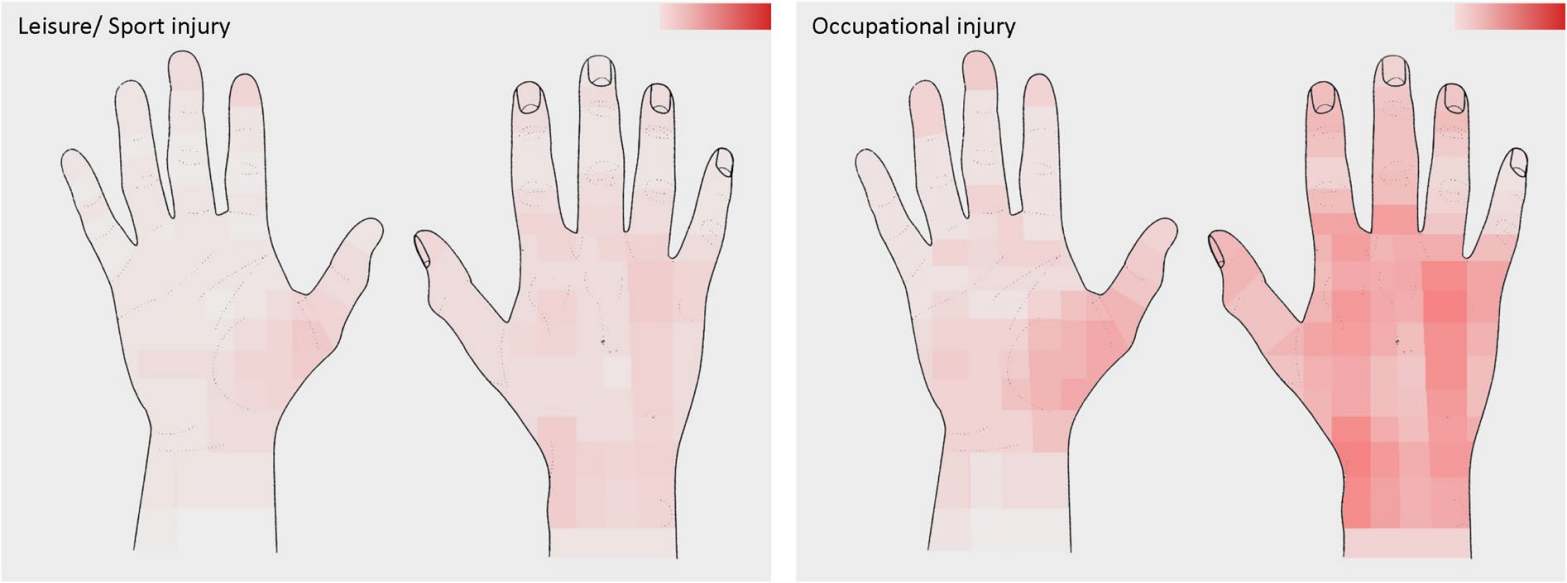
Heatmaps with color-graded frequency depicting the location of injury for leisure/sports injury and occupational injury. The higher the color intensity, the higher the frequency

**Fig. 4:**
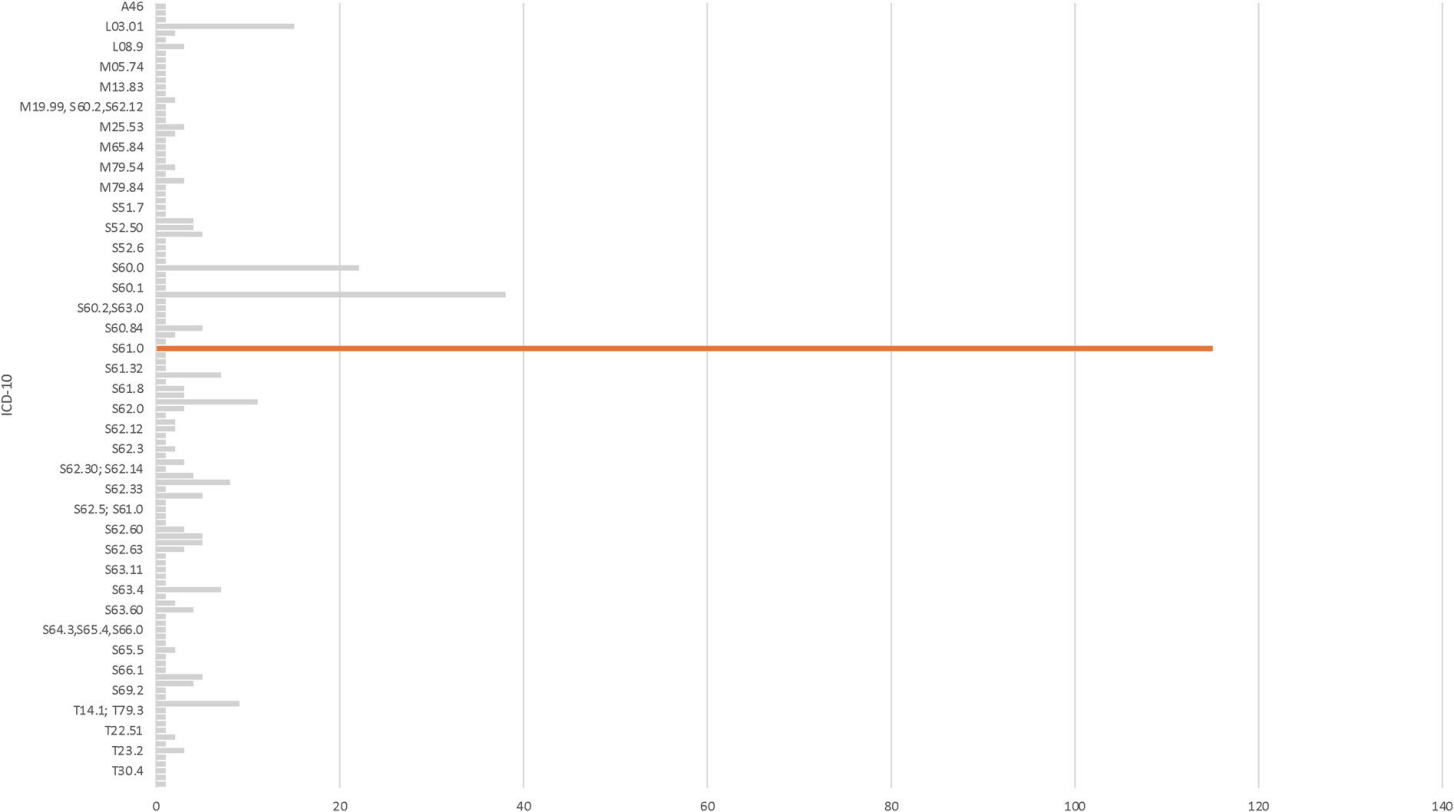
Bar graph depicting the diagnosis of patients presenting to the emergency department with hand injury classified according to the International Classification of Diseases (ICD-10). Data presented as absolute frequencies. Most common diagnosis included: S61.0: an *open wound of finger(s) without damage to nail*; S60.2: a *contusion of other parts of wrist and hand*; S60.0: the *contusion of finger(s) without damage to nail*; L03.01: *Cellulitis of finger*; S61.9: *Open wound of wrist and hand, part unspecified*

Table 1 summarizes data related to the trauma mechanism. While most patients were right-handed (91%, *n* = 295/324), both the left and the right hand were injured with similar frequency (left: 43%, *n* = 191/417; right: 50%, *n* = 217/417; both: 2%, *n* = 9/417). Leisure or sport injuries were reported most commonly (75%, *n* = 216/289), followed by occupational trauma (17%, *n* = 73/289). The fingers (Dig. II–V) were injured most frequently (43%, *n* = 171/402), followed by metacarpals (19%, *n* = 76/402) and the thumb (14%, *n* = 56/402). Blunt trauma and cuts accounted for the majority of injuries (74%, *n* = 303/411). Hand-graphics (Figs. 2, 3) show color-coded frequencies of the affected areas of the palmar and dorsal aspect of the hand for the most common types of injury (blunt injury, cuts), as well as the most frequent circumstances of accident (occupational/leisure and sport). Diagnoses upon discharge were classified according to ICD-10 and are presented in Fig. 4 and Table 2. The ICD-10 code S61.0 was the most frequent diagnosis, referring to an *open wound of finger*(*s*)* without damage to nail*, followed by S60.2, a *contusion of other parts of wrist and hand*, and S60.0, the *contusion of finger*(*s*)* without damage to nail*.

**Table 1:** Trauma-related data of the study population

Variable	*N*	%
Circumstance of accident (*n* = 289)		
Occupational	73	25.3
Leisure or sport	216	74.7
Injured hand (*n* = 417)		
Right	217	52.0
Left	191	45.8
Both	9	2.2
Localization of injury (*n* = 402)		
Thumb	56	13.9
Finger (II–V)	171	42.5
Metacarpal	76	18.9
Wrist	44	10.9
Forearm	4	1.0
Multiple	51	12.7
Type of injury (*n* = 411)		
Blunt injury	161	39.2
Cut	142	34.5
Crush	16	3.9
Needle stick	10	2.4
Infection	15	3.6
Burn	7	1.7
Bite	10	2.4
Amputation	4	1.0
Other	33	8.0
Multiple	13	3.2

Data are presented as absolute and relative frequencies

**Table 2:** Diagnoses upon discharge classified according to the International Classification of Diseases (ICD-10)

ICD-10	*N*	ICD-10	*N*	ICD-10	*N*	ICD-10	*N*
S61.0	115	S61.80	3	S51.9	1	S52.8	1
S60.2	38	S63.6	2	S63.08	1	S62.2	1
S60.0	22	S62.3	2	S61	1	S63.62	1
L03.01	15	L03.10	2	S63.12,S63.4	1	S62.23	1
S61.9	11	S60.9	2	M13.14	1	S64.3,S65.4,S66.0	1
T14.1	9	M79.54	2	M79.63	1	S52.59	1
S62.32	8	M19.83	2	S61.0; S60.81; S60.2	1	S64.8, S61.80	1
S63.4	7	S65.5	2	S64.4, S66.0	1	S62.3; S62.6	1
S61.7	7	S62.1	2	S61.3	1	S65.5,S62,61,S68.1	1
S62.61	5	T23.0	2	S66.0	1	M24.24	1
S52.51	5	S62.12	2	S61.32	1	S66.1	1
S62.62	5	M65.03	2	S69.2	1	S62.30; S62.14	1
S60.84	5	M79.94	1	M13.83	1	M79.84	1
S62.34	5	S63.52	1	T14.3	1	M25.49	1
S66.3	5	M67.43	1	S61.7,A46	1	S69.8	1
S63.60	4	S60.1	1	T23.1	1	L0.22	1
S52.50	4	S56.10	1	M18	1	T14.1; T79.3	1
S68.1	4	M05.74	1	T30.4	1	S62.33	1
S62.31	4	T23.20	1	A46	1	T22.11	1
S52.5	4	S60.2, S60.0	1	M65.84	1	S51.7	1
S61.8	3	S63.11	1	L03.10, S61.9	1	S60.0; S61.0	1
M79.64	3	S60.2,S63.0	1	M67.44	1	S62.4	1
L08.9	3	S63.68	1	M19.99, S60.2,S62.12	1	Q68.1	1
T23.2	3	S60.81	1	S63.10	1	S62.5; S61.0	1
M25.53	3	M 34.0	1	S62.04	1	T30.2	1
S62.0	3	S60.83	1	S63.12	1	S62.52	1
S62.60	3	T22.51	1	L02.4,T80.2	1	T79.3	1
S62.63	3	M10.04,M61.4	1	S52.6	1	S62.6	1
S62.30	3	T81.2	1	L97	1	S60.0; S62.63	1
						M65.14	1

Data presented as absolute frequencies. Most common diagnosis included: S61.0, *an open wound of finger(s) without damage to nali*; S60.2, *a contusion of other parts of wrist and hand*; S60.0, *the contusion of finger(s) without damage to nail*; L03.01, *Cellulitis of finger*; S61.9, *Open wound of wrist and hand, part unspecified*

### Clinical examination and diagnostic measures

**Fig. 5:**
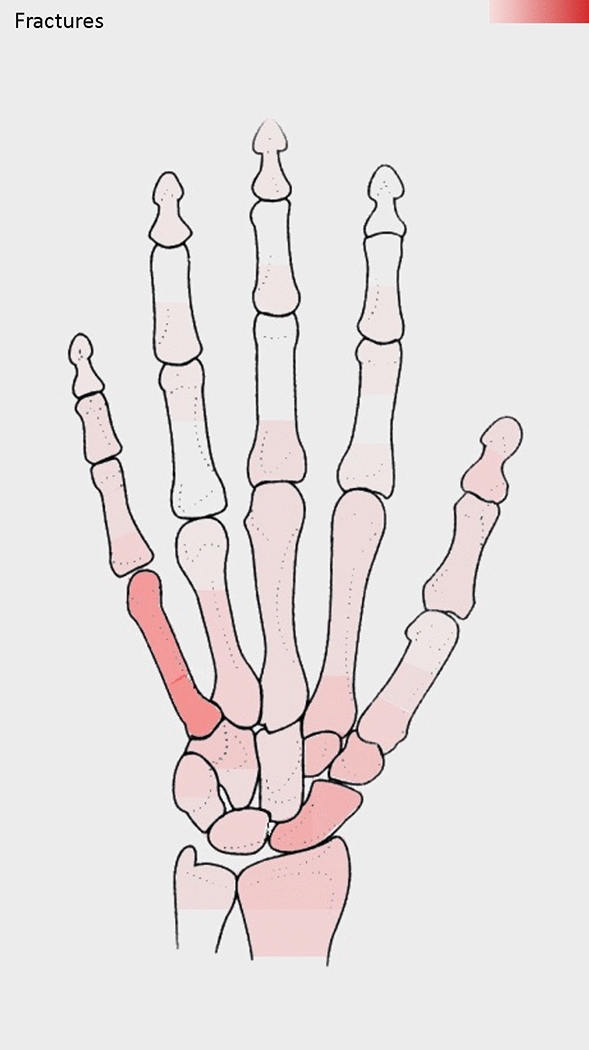
Heatmap with color-graded frequency depicting the location of fractures. The higher the color intensity, the higher the frequency

Detailed data regarding the clinical examination and diagnostics are tabulated in Table 3. The mean level of pain reported on a visual analogue scale (0–10) was 3.76 ± 2.36. Initial clinical exploration most frequently revealed superficial wounds without damage to imperative anatomic structures (46%, *n* = 63/138). Injury of the bone upon clinical exploration was found in a third of all cases (30%, *n* = 41/138), with fractures reported in 23% of cases (*n* = 66/286) after radiological examination. The most common fracture location was the 5th metacarpal, as depicted in Fig. 5. Of the radiological assessments performed, X-ray imaging was utilized most often (93%, *n* = 238/255), followed by computed-tomography (7%, *n* = 17/255).

**Table 3:** Clinical examination and diagnostics

Variable	*N*	%
Affected structures (*n* = 138)		
Superficial wound	63	45.7
Bone	41	29.7
Extensor tendon	7	5.1
Flexor tendon	3	2.2
Joint	8	5.8
Nerve	2	1.4
Muscle	1	0.7
Vessel	2	1.4
Multiple	11	8.0
Fracture (*n* = 286)		
Yes	66	23.1
No	220	76.9
Medical imaging (*n* = 255)		
X-ray	238	93.3
Computed-tomography	2	0.8
Both	15	5.9

Data are presented as absolute and relative frequencies

### Therapeutic measures

Detailed data regarding therapeutic measures are summarized within Table 4. A third of all patients required a tetanus booster injection (30%, *n* = 71/239). Splint immobilization was reported in *n* = 141 patients. Of these, 18% (*n* = 26) were fixed in intrinsic plus position, 11% (*n* = 16) were wrist circular casts and 6% (*n* = 9) were mallet-finger splints, while 64% (*n* = 90) were not further specified. Emergent surgical intervention was reported in 74 patients (27%, *n* = 74/277). Elective surgery was recommended in 91 cases (25%, *n* = 91/357). Follow-up treatment within the in-house department for hand surgery was recommended in over 50% of cases post discharge from the emergency department (51%, *n* = 150/296).

**Table 4:** Treatment initiated within the emergency department

Variable	*N*	%
Tetanus protection (*n* = 239)		
Yes	168	70.3
No	71	29.7
Splint immobilization (*n* = 141)		
Intrinsic plus	26	18.4
Mallet-finger	9	6.4
Wrist circular cast	16	11.3
Unspecified	90	63.8
Elective surgical procedure (*n* = 357)		
Yes	91	25.5
No	266	74.5
Emergency procedure (*n* = 277)		
Yes	74	26.7
No	203	73.3
Follow-up treatment post discharge (*n* = 296)		
Office-based physician/general practitioner	95	32.1
University hospital hand surgery	150	50.7
Other university hospital facility	5	1.7
Emergency department	46	15.5

Data are presented as absolute and relative frequencies

## Discussion

The aim of this study was to determine injury patterns, frequency, severity and localization of injury in hand trauma patients who referred to a single-center surgical emergency department of a level 1 hospital in Germany. In addition to this, the study aimed to depict differing situational causation of injuries to the hand.

The collected data portrays that the majority of hand injuries occur during leisure time activities. According to statistical analysis from the Federal Institute for Occupational Safety and Health of Germany, it is confirmed that accidents occurring during leisure time or at home, account for as much as 72% (7.05 million) of a total of 9.73 million injuries [29]. These findings are confirmed by studies from Austria, where work accidents accounted for 14.7% (*n* = 117,148), road and traffic accidents for 10.3% (*n* = 81,900) of all injuries, while household accidents alone accounted for 39% (*n* = 306,800) and leisure accidents including sports accidents for 36.3% (*n* = 288,800) of all injuries. Thus, 75% (*n* = 595,600) of the patients recorded were injured in their home, during recreational activities, or during sports [30].

Hand injuries can result in significant disability limiting participation in societal activities and work. An evaluation of hand trauma in France for the year 2008 showed that 35% of injuries resulted in incapacity for work [31]. The number of all days of absence from work due to hand injuries totalled at 5,500,000. The importance of recording the epidemiological differences throughout this data is imperative in order to highlight the relevance of improvement of preventive measures and allocation of health care services and thus, to reduce the socioeconomic costs of medical care and sick days due to incapacitation.

### Gender distribution and age

From the data, it appears that the "typical patient" presenting to the emergency department with hand injury is a young male. The male-to-female sex ratio was approximately 2 to 1, which was very similar to that of Hill et al. who analyzed data from 4873 hand and wrist injuries of Northern Ireland’s Trauma Centers (2.2:1) or Butala et al. (1.5:1) from the U.S. who described 1147 cases from a Level 1 trauma center [32, 33]. Similarly, in a large study conducted in emergency departments in North America, upper extremity injuries were seen in male patients predominantly [34]. The mean patient age of the study population in this investigation was 39.46 ± 17.69 years which is comparable to the literature [34–36]. However, data can vary, as studies reporting sports injuries show younger study populations [21, 24], compared to those reporting work-related injuries exclusively [19]. In clinical practice, physicians need to assess and manage patients from childhood, over adolescence to old age. This is highlighted by the broad range of age in this study population (16–90 years). Unfortunately, data for patients < 16 years of age were not included in this study, as they were primarily referred to a children´s hospital. Nonetheless, coping with young children is even more challenging and must not be underestimated. An analysis of circumstance and type of injury with regard to patient age was outside the scope of this study, but could provide further important data in the future.

### Color-coded heatmaps

Previously, color-coded heatmaps have proven to be a useful tool in visualizing the skin surface pain projection of common wrist pathologies [28]. Here, the use of heatmaps was expanded, as they were utilized to mark the frequencies of the affected areas of the palmar and dorsal aspect of the hand for the most common types of injury (blunt injury, cuts), as well as the most frequent circumstances of accident (occupational/leisure and sport). Interestingly the heatmaps demonstrate that the dorsal aspect of the hand seems to be more vulnerable to injury, compared with the palmar hand. In addition, the radial wrist and thenar region on the palmar and dorsal aspect, as well as the fingertips of the second and third finger appear to be markedly exposed to trauma. In the future, heatmaps need to be created for more specific injury types to derive further clinically relevant data, in order to further define “anatomic danger zones” using color-coded heatmaps. This method of data visualization goes beyond the possibility of ICD coding of injuries and provides more detailed and clinically relevant information on injury patterns.

### Diagnostic work-up

Radiological imaging was reported in 255 cases, which accounts for over 50% of all cases. However, a fracture was reported in only 66 patients. Hence, one could argue that clinical examination ought to be improved, in order to reduce the number of radiographs performed. As a matter of fact, young physicians often perform rotations in the emergency department at the beginning of their residency. They lack clinical experience which may play a role in contributing to unwarranted diagnostic measures such as radiological imaging being performed. Improvement of training, guidance and consulting with experienced staff is therefore mandatory to help in the diagnostic process and minimize unnecessary radiation. Even though fractures were a frequent diagnosis in the emergency department according to the data, there were merely three patients specifically reported with scaphoid fractures (*n* = 3 patients with an ICD-10 diagnosis S62.0—Fracture of navicular [scaphoid] bone of hand vs. *n* = 60 patients with a diagnosis within the group of S62—Fracture at wrist and hand level). Possibly, this points towards the fact that these patients are often not seen primarily in the emergency department, due to unfortunate trivialization of the injury. Instead, patients are more likely to be seen in the hand surgical outpatient clinic after secondary referral from external colleagues. There is discrepancy with regard to the number of fractures reported (*n* = 66), when compared to the ICD-10 diagnosis at discharge including fractures at the wrist and hand level (S62 group, *n* = 60), in addition to distal radius fractures (S52 group, *n* = 16). This highlights the need for an improved documentation system and controlling mechanisms in order to gather high-quality data.

The importance of training residents of all surgical disciplines working in emergency care in the basics of hand surgical examination needs to be further emphasized. Although clinical examination revealed a “superficial wound” without damage of any imperative anatomic structures, over 50% of patients were further referred to the hand surgery outpatient clinic. Possibly, this is also related to the insufficient training of residents in surgical emergency departments in hand surgical examination. Referral to hand surgical specialists after diagnosis of a superficial wound might reflect potential insecurity in the nature of the diagnosis. This referral of every second patient to specialist hand surgical examination underlines that appropriate diagnostics and management of hand injuries is of great importance, as underestimated or missed injuries can lead to lifelong disability with consequent loss of work and of quality of life.

### National and international lack of data

To date, there is a lack of standardized guidelines for documenting hand injuries in an acute setting [37, 38]. Due to the insufficient documentation and classification by means of an international score, data on hand injuries are not readily available in order to perform detailed epidemiological analysis, investigating affected structures, patterns of injuries or their localizations. In Germany, there have been recent efforts to homogenize reporting of hand injuries on a national level. The German Society for Hand Surgery (“Deutsche Gesellschaft für Handchirurgie”—DGH) introduced a national Hand-Trauma-Registry in 2018. It is an initiative to record (severe) hand injuries in a standardized manner. Aim is to gain clinically significant outcome data, and to provide a database for monitoring the structure and quality of care. The first annual report of this registry was published in February 2021 [39]. In the future, this database will further flourish and provide high-quality multi-center data that can be utilized to determine associations between patient characteristics, trauma mechanisms, the localization of injuries and injured structures using multivariate analysis. However, in this registry, only those cases that require operative care are collected. Relevant epidemiological data on patients which need outpatient treatment only could therefore be lost.

### Limitations

The data were collected from a single-center emergency department. Only patients with acute trauma presenting to the emergency department were included, as opposed to patients seen at hand-surgery outpatient clinics. Unfortunately, data entry into the ‘Hand Trauma Documentation’ sheet was not monitored or controlled. This can be considered the greatest limitation of the study, as there are missing data in several outcome parameters. In addition, one might argue that physicians prefer to document actual findings, such as injured structures, or the requirement for surgical intervention, as opposed to conservative treatment or documentation of the lack of any injury. Thus, some findings might be underreported, while others might be overreported which might skew the analyses. In the setting of an interdisciplinary surgical emergency department, with significant patient numbers across numerous subspecialties, loss of data needs to be accounted for. In addition, data were obtained using handwritten documentation forms. A digital documentation system was recently installed, which will allow for higher quality data in the future.

## Conclusions

In this study, epidemiological information on hand injuries and their patterns is presented based on a standardized “Hand-Trauma Documentation” form utilized for the assessment of hand trauma within a single-center emergency department. In addition, a method to visualize affected areas on the palmar and dorsal aspect of the hand is provided by introducing the use of heatmaps that are based on color-coded frequencies. The dorsal aspect of the hand including the 5th metacarpal, the radial wrist and thenar region, as well as the fingertips of the second and third finger are markedly exposed to trauma and represent anatomic danger zones to injury of the hand. Overall, the data supports the requirement for specific training in hand surgical examination for all surgical specialties working in emergency care, in order to increase the quality of diagnostic work-up and management of injuries of the hand. In addition, this study supports further action regarding the formulation of preventative measures to reduce the incidence of recreational and work-related injury of the hand.

## Supplementary Information

Below is the link to the electronic supplementary material.

Supplementary file1 (TIFF 3868 KB)
