# Supplementary figures and images for: Epidemiology of hand injuries that presented to a tertiary care facility in Germany: a study including 435 patients
Source: Arch Orthop Trauma Surg. 2022 Sep 22;143(3):1715–24. doi: 10.1007/s00402-022-04617-9 (PMC9958136; doi:10.1007/s00402-022-04617-9)

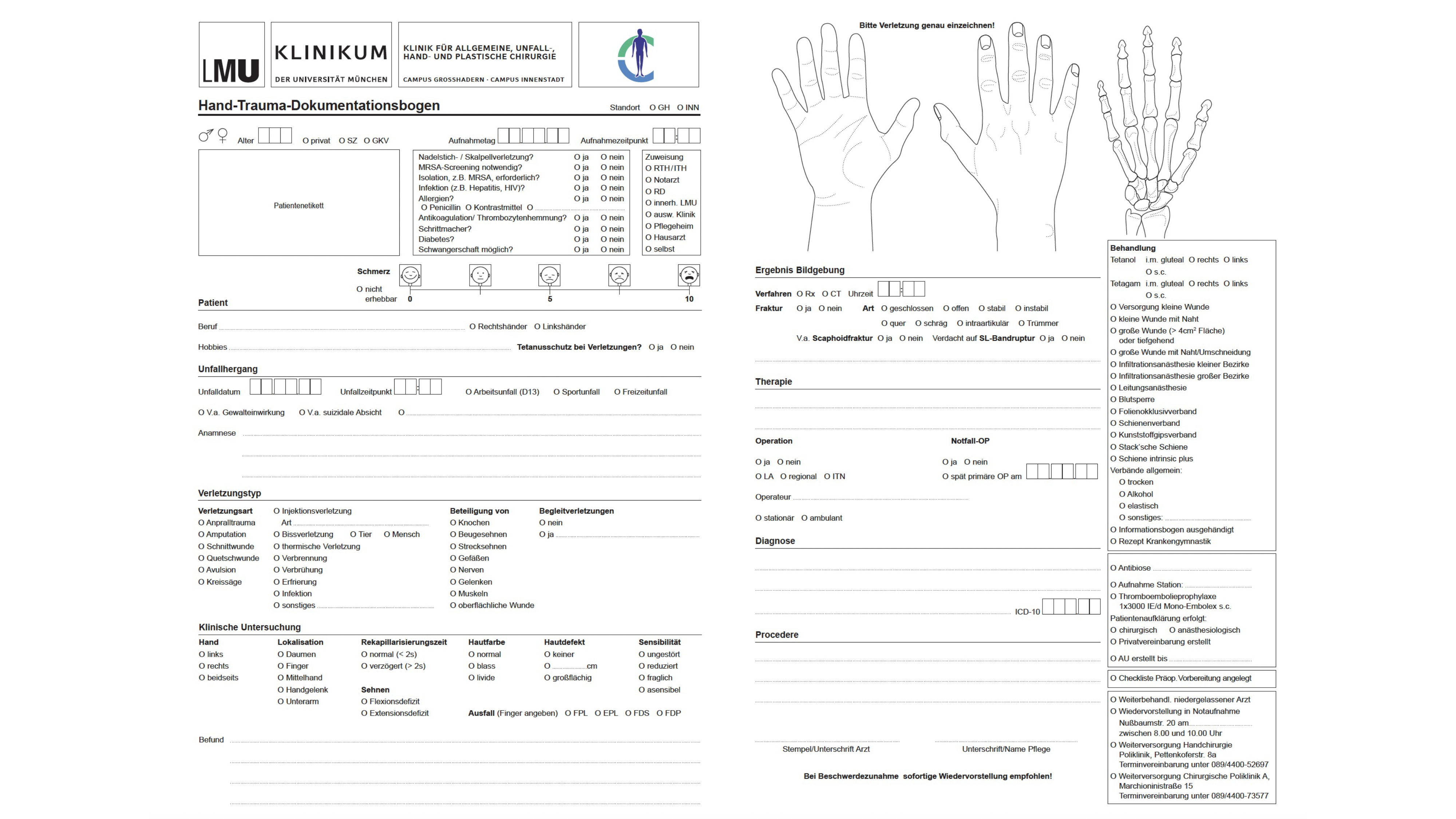

Supplement: Supplementary file 1 — Supplementary file1 (TIFF 3868 KB) [file 402_2022_4617_MOESM1_ESM.tiff]
